# Association between leafy vegetable consumption and incidence of metabolic syndrome and its symptoms: a systematic review of prospective cohort and randomised control trials

**DOI:** 10.1007/s00394-025-03750-6

**Published:** 2025-07-05

**Authors:** Esther N. Muriuki, Begum Celik, Gunter G. C. Kuhnle, Charlotte E. Mills

**Affiliations:** 1 Department of Food and Nutritional Sciences, University of Reading, Whiteknights, Harry Nursten Building, Reading, RG6 6AP UK; 2 Department of Food Science, Meru University of Science and Technology, Meru-Maua Road, Meru, Kenya

**Keywords:** Metabolic syndrome, Leafy vegetable, Systematic review

## Abstract

**Purpose:**

Metabolic syndrome (MetS) is a combination of cardiometabolic risk factors that burden health systems worldwide. Vegetables contain substances that are essential in preventing chronic illnesses. However, the effect of leafy vegetable consumption on the incidence of MetS and its symptoms is unclear. We systematically reviewed the association between leafy vegetable consumption and the incidence of MetS and its symptoms.

**Methods:**

The Web of Science, Scopus, and MEDLINE databases were searched for relevant publications until August 2023. Randomised control trials (RCT) and prospective cohort studies examining the effect of consumption of leafy vegetable/s on MetS or its components in healthy adults were included.

**Results:**

Ten studies (eight RCTs and two cohort studies) were included. A reduction in systolic blood pressure after intervention with leafy vegetables was observed in one RCT, but no effect on blood pressure was reported in two RCTs. One cohort study reported reduced hypertension incidence with increased leafy vegetable intake. A reduction in blood glucose was observed in three RCTs, and two RCT found no change. One cohort study reported no association between leafy vegetable consumption and the incidence of type 2 diabetes. Two RCTs found no change in blood lipids. No trials assessing leafy vegetable consumption and obesity or MetS were found.

**Conclusion:**

Few studies evaluate the impact of leafy vegetable consumption on MetS and its symptoms. Beneficial effects are reported for blood glucose and blood pressure regulation, but the evidence is limited. More studies are needed to build a robust body of evidence.

**Supplementary Information:**

The online version contains supplementary material available at 10.1007/s00394-025-03750-6.

## Introduction

Metabolic syndrome (MetS) is a cluster of cardiovascular disease risk factors occurring together, which include three or more of the following: central obesity; men  ≥ 85 cm, women ≥ 80 cm, or body mass index (BMI)  >  30 kg/m^2^, elevated fasting blood glucose ≥ 5.6 mmol/L, high blood pressure ≥ 130/85 mmHg, elevated blood triglycerides ≥ 1 .7 mmol/L and low high-density lipoprotein (HDL) < 1.0 mmol/L [1–4]. The precise prevalence of MetS worldwide is difficult to determine due to overlapping definitions. The prevalence estimates range from 4 to 10% [5] worldwide, 13%–36%, and 27.9–32.5% in Europe and in the United States respectively [6–9]. Most people with type 2 diabetes also have MetS [2], increasing the prevalence estimate to over a billion people [10]. This high prevalence of MetS and its symptoms significantly burden health systems globally. MetS also causes a twofold increase in cardiovascular disease (CVD) risk and a fivefold increase in type 2 diabetes [1, 11]. Each MetS risk factor poses a significant risk of CVD, which increases with the number of symptoms present.

Diet is a modifiable risk factor that can support the prevention of MetS. of the dietary approaches, consuming fruits and vegetables has been shown to protect against many chronic illnesses. For example, the World Health Organisation (WHO) recommends consuming at least five portions of fruits and vegetables daily to protect against chronic diseases [12]. The Mediterranean diet, composed of high quantities of fruits and vegetables, is inversely associated with cardiovascular disease risk [13]. The Dietary Approaches to Stop Hypertension (DASH) diet is recommended to prevent and manage hypertension [14]. Fruits and vegetables are often classified as a single dietary component despite significant differences in composition; this difference in composition could have varied health impacts.

Different studies have found varying associations between individual groups of fruits and vegetables. [15–18], suggesting that consuming leafy vegetables may confer greater benefits than other vegetables [15, 16, 19–24]. Leafy vegetables form a large subgroup of vegetables, including a wide range of vegetables such as spinach, kale, cabbage and lettuce. These vegetables are rich in micronutrients and contain bioactive compounds that are protective against chronic illnesses [23, 25, 26]. However, it is unknown whether the vegetables themselves could protect against MetS, although it is biologically plausible. For example, nitrates in green vegetables increase the bioactive NO supply and improve endothelial function, which helps reduce blood pressure [27, 28]. Flavonoids increase NO circulation, resulting in higher flow-mediated dilation and blood pressure reduction [29–31]. In addition, dietary fibre forms viscous gels in the stomach, which slow gastric emptying and attenuate postprandial blood glucose and lipids. Fibre also improves satiety, prevents weight gain, and increases insulin sensitivity [32]. Leafy vegetables are low in energy and have a low glycemic index, which is effective in controlling overweight and obesity [33–35].

Previous reviews that have combined fruits and vegetables as a single dietary component observed an inverse association between consumption of fruits and vegetables and MetS [14, 36, 37]. Some reported that this positive effect is due to fruits and not vegetables [38, 39]. Others have suggested a synergistic effect [40]. However, very few studies have investigated the protective effect of consuming leafy vegetables as a single food component on the incidence of MetS or incidence of its symptoms. Understanding this could support specific guidelines for prevention of MetS. Notably, most existing studies involves participants with underlying health conditions, focusing on disease management rather than prevention in healthy population. Therefore, we conducted a systematic review of randomised control trials (RCT) and prospective cohort studies to investigate if the consumption of leafy vegetables is associated with a reduced incidence of MetS or its symptoms. We included prospective cohort studies to evaluate whether the prolonged consumption of leafy vegetables reduces the incidence of MetS while RCTs were included to evaluate acute or short-term changes in parameters associated with the onset of MetS. By focusing on healthy individuals, we aimed to assess the preventive potential of leafy vegetables before the onset of metabolic abnormalities, ensuring that any observed effects were directly attributable to the intervention and not confounded by pre-existing conditions.

## Methods

This systematic review was conducted in accordance with the PRISMA (Preferred Reporting Items for Systematic Reviews and Meta-Analyses) guidelines [41]. This study was registered in the International Prospective Register of Systematic Reviews (PROSPERO), CRD42020201925.

### Eligibility criteria for this review

#### Types of studies

Randomised control trials (RCT) and prospective cohort studies were included. Retrospective cohort, cross-sectional, and case–control studies were excluded. We included studies published in the English language. Other details on the eligibility criteria are listed in Table 1**.**

**Table 1:** Eligibility criteria for inclusion of studies to the review

	Randomised control trials	Cohort studies
Population	Studies with participants aged ≥ 18 years were included Studies were excluded if the participants had a chronic illness or any of the following MetS symptoms before enrolment: Central obesity: waist circumference ≥ 94 cm in men and ≥ 80 cm in women Obesity: BMI > 30 kg/m^2^ High blood pressure: systolic > 130 mmHg diastolic > 85 mmHg Dyslipidaemia: triglycerides ≥ 1.7 mmol/L, HDL cholesterol < 1.0 mmol/L in men and < 1.3 mmol/L in women Raised fasting blood glucose: ≥ 5.6 mmol/L or type 2 diabetes ANIMAL studies were excluded	Studies with participants aged ≥ 18 years were included Studies were excluded if the participants had a chronic illness or any of the following MetS symptoms before enrolment: Central obesity: waist circumference ≥ 94 cm in men and ≥ 80 cm in women Obesity: BMI > 30 kg/m^2^ High blood pressure: systolic > 130 mmHg diastolic > 85 mmHg Dyslipidemia: triglycerides ≥ 1.7 mmol/L, HDL cholesterol < 1.0 mmol/L in men and < 1.3 mmol/L in women Raised fasting blood glucose: ≥ 5.6 mmol/L or type 2 diabetes ANIMAL studies were excluded
Intervention	Any leafy vegetable It must have included the consumption of the whole leaf and not a filtrate or an extract Studies were excluded if the intervention was a herb or plant used primarily for medicinal purposes, and not as part of the common diet	Studies where the exposure was consumption of leafy vegetable/s over a period of time were included Studies involving herbs or medicinal plants were excluded
Comparator	Intervention without leafy vegetables	Habitual diet, or increased intake of leafy vegetables versus a low leafy vegetable intake
Outcome measures	Studies were included if changes in the following outcomes were measured: Fasting/postprandial blood glucose Blood pressure Blood triglycerides HDL cholesterol Body mass index Waist circumference Number of MetS symptoms	Studies that assessed the incidence of MetS or the incidence of individual MetS symptoms as previously defined

MetS, metabolic syndrome; BMI, body mass index; HDL, high density cholesterol

#### Information sources and search strategy

Three databases, Web of Science, MEDLINE, and Scopus, were searched from inception to 08th August 2023. With the help of a librarian, we designed a search strategy with various keywords related to leafy vegetables, MetS, and its symptoms. The keywords included ‘metabolic syndrome,’ ‘leafy vegetable,’ ‘blood pressure,’ ‘blood glucose,’ ‘triglyceride,’ ‘HDL cholesterol,’ and ‘obesity’. The search strategy is available in the supplementary material Table 1. We limited our searches to journal articles published in the English language.

#### Selection process

Articles were downloaded to Endnote reference manager version X9. The duplicates were removed, and two reviewers, ENM and BC, screened the titles and abstracts of the articles. Each reviewer screened the articles independently. The two lists were compared, any discrepancies were discussed, and a decision was made. Finally, ENM and BC screened the full text of the eligible articles, and where there was disagreement on the eligibility of papers, the articles were discussed with GGCK and CEM, and a decision was made.

#### Data collection process

A form was designed in Excel format and was used by reviewers (ENM and BC) to collect data from eligible studies. ENM extracted the data, and BC double-checked the information for correctness. In studies with missing data, we contacted the author of the study.

#### Data items

Articles containing RCT and cohort studies were separately categorised according to the outcome. Any study reporting a change in any MetS symptoms was eligible for inclusion. For blood glucose, we included studies that reported a change in fasting blood glucose, postprandial blood glucose, or the incidence of type 2 diabetes. In addition, we included studies that reported changes in either systolic or diastolic blood pressure or both or the incidence of hypertension. For central obesity, studies that reported changes in waist circumference, BMI, or incidence of central obesity or obesity were eligible. In addition, studies were eligible if they measured changes in triglycerides, HDL cholesterol, or incidence of dyslipidaemia. In studies that described the participants as healthy before enrolment, we assumed that the participants did not have any of the MetS symptoms.

The following data was extracted for each study: author/s of the article, year of publication, country of study, number of participants, age, gender and health status of participants, type and amount of leafy vegetable used for intervention, duration of the intervention, the method used to assess the outcome. Outcomes measures presented in graphical form were digitised using the Plot Digitizer web software to extract and estimate numerical values from the plotted graphs [42].

### Assessment of risk of bias in included studies

Two reviewers, EM and BC, assessed the risk of bias in the included studies. For the RCTs, we used the Cochrane risk of bias tool. This tool has the following domains: (1) Randomisation process, (2) Bias arising from period and carryover effects, (3) Deviations from the intended interventions, (4) Missing outcome data, (5) Measurement of the outcome, (6) Selection of the reported result. Based on the items, the studies were categorised as high, medium, or high risk of bias. We used the Newcastle–Ottawa Scale to evaluate the risk of bias in cohort studies; three main items were considered: The selection, comparability of the cohorts and the assessment of the outcome. maximum number of points for each item was 4, 2, and 3, respectively, with a maximum of 9 points per study. Studies with 0–3, 4–6, and 7–9 were described as having a high, medium, and low risk of bias, respectively.

### Data synthesis

Since meta-analysis was impractical due to the limited number of included studies, forest plots were created using the metafor package in R version 4.3.2 to visually summarise the effect sizes and 95% confidence intervals of individual RCTs [43, 44].

## Results

### Study selection

**Fig. 1:**
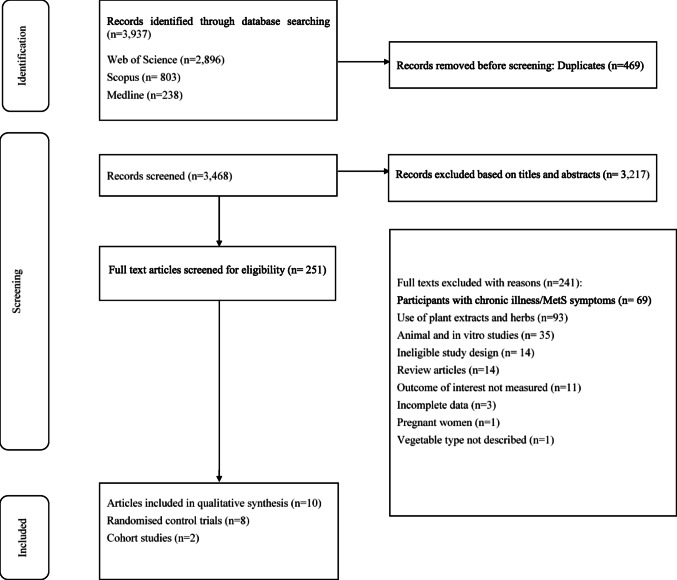
PRISMA flow diagram detailing the search and selection process

The PRISMA flow diagram (Fig. 1) details the search and selection process. A total of 3937 articles were retrieved, of which 469 duplicates were removed. The title and abstract of the remaining 3468 articles were screened, and 3217 articles were excluded. The full text of 251 articles were screened; after exclusion (n = 241), ten studies remained for inclusion in the analysis [45–54]. We contacted the authors of two studies [55, 56] because there was missing data on the study outcomes; since the authors did not respond, we excluded the studies.

### Study characteristics

A summary of the ten included study characteristics is presented in Tables 2 and 3. We included eight RCTs (three chronic trials and five acute trials) [45, 47–49, 51, 52, 54] and two cohort studies [46, 50]. The articles were published from 2013 to 2021 and were conducted in Australia, India, Iran, Japan,Pakistan, Singapore, and the USA. The number of participants in the RCTs ranged between 10 and 90, aged between 20 and 70 years, and without MetS symptoms. The cohort study participants were 1544 and 48,371, aged between 26 and 75 years at baseline.

**Table 2:** Characteristics of studies examining the association between leafy vegetable consumption and blood pressure

References	Country	Study design	Duration of study	Sample size	Gender	Age (years)	Intervention/exposure	Dose	Results
Golzarand et al. (2016) [46]	Iran	Cohort Study	3 years	1544	880 women, 664 men	26–50	Increased intake of leafy vegetable	427 g/day in the highest quartile	Reduced incidence of hypertension with increased intake
Mayra et al. (2019) [51]	USA	Chronic RCT	10 days	10	women	52–57	Leafy vegetable salad	80 g/day	No significant change in blood pressure
Bondonno et al. (2014) [57]	Australia	Chronic RCT	7 days	38	12 men, 26 women	38–70	Leafy vegetable salad	370 g/day	No significant change in blood pressure
Liu et al. (2013) [49]	Australia	Acute RCT	3.5 h	26	6 men, 20 women	38–69	Spinach	250 g cooked	A decrease in systolic blood pressure and no effect on diastolic blood pressure

**Table 3:** Characteristics of studies examining the association between leafy vegetable consumption and blood glucose

References	Country	Study design	Duration of study	Sample size	Gender	Age (years)	Intervention/exposure	Dose	Results
Kurotani et al. (2013) [50]	Japan	Cohort study	5 years	48,437	21,269 men, 27,168 women	45–75	Leafy vegetable intake	47.2 g/ day in the highest quartile	No significant association
Kushwaha et al. (2015) [45]	India	Chronic RCT	3 months	90	Postmenopausal women	45–60	Amaranth or moringa leaves	9 g/ day dried	Reduced fasting blood glucose for both amaranth and moringa leaves
Shokraei et al. (2021) [58]	Iran	Acute RCT	4 h	16	Men	20–30	Watercress or lettuce	100 g raw	Decrease in postprandial blood glucose for lettuce and no change for watercress
Ahmad et al. (2018) [54]	Pakistan	Acute RCT	2 h	20	10 men, 10 women	23–25	Moringa leaves	5 g dried	No significant change in postprandial glucose
Sun et al. (2014) [48]	Singapore	Acute RCT	3 h	12	6 men, 6 women	21–50	Bok choy	120 g boiled	Decrease in postprandial blood glucose
Maruyama et al. (2013) [47]	Japan	Acute RCT	2 h	10	Men	20–35	Spinach	100 g boiled	No significant change in postprandial glucose

The study participants were women and men only in two chronic and two acute RCTs, respectively, while four RCTs recruited both men and women. The interventions used in the RCTs were leafy vegetable salad (n = 2), spinach (n = 2), moringa leaves (n = 2) watercress and lettuce (n = 1), bok choy (n = 1), and amaranth (n = 1). The evaluated study outcomes in the RCTs were blood pressure in three studies, blood glucose in three studies, and blood glucose and triglycerides in two studies. The length of the intervention was 10–90 days in the chronic RCTs and 2–4 h in the acute RCTs. The four acute RCTs used a crossover study design, while the parallel (n = 1) and crossover design (n = 3) were used in the chronic RCTs.

In the two included cohort studies, participants included both men and women. The follow-up period was 3 and 5 years. The assessed exposure was the consumption of an assortment of leafy vegetables expressed as grams per day and fitted in quantiles. The assortment contained spinach in all the studies. The incidence of MetS symptoms was compared in the participants with a high intake of leafy vegetables against those with a low intake. The outcomes assessed were the incidence of type 2 diabetes in two studies and hypertension in one study.

## Study quality

### Risk of bias for the RCT studies

A summary of the risk of bias in the studies is shown in Supplementary Table 2 and Supplementary Table 3. Five RCTs [48, 49, 51, 57, 58] had a medium risk of bias, while two [45, 47] had a high risk of bias. Only one chronic RCT [57] used a computer-generated randomisation method but did not describe how the allocation sequence was concealed from the participants. In four acute RCT studies [48, 49, 54, 58], the authors described the studies as randomised but did not describe the randomisation process. In the RCTs by Maruyama et al. [47] and Kushwaha et al. [45], the studies were not described as randomised, nor was the process of randomisation described, and the allocated interventions were not concealed from the participants nor the investigators; therefore, the studies had a high risk of bias.

In all the studies, the investigators and the participants were aware of the intervention they were receiving, and no details were given as to whether the persons who measured the outcomes were blinded.

There were no deviations from the intended interventions, all participants were accounted for from beginning to completion of the study, and there was no participant dropout in all the studies; hence, none of the studies had a risk of reporting bias.

### Risk of bias for the cohort studies

One cohort study [46] had a low, and one had a medium risk of bias [50]. In the study by Golzarand et al.[46], all participants were selected through random cluster sampling and followed up from the beginning to the end of the study. A trained physician ascertained hypertension and the study was adjusted for all the parameters that could have impacted blood pressure. The study by Kurotani et al. [50] had a medium risk of bias because the participants self-reported the incidence of type 2 diabetes. The study did not describe how the recruited participants were followed up to the end of the trial.

### Association between consumption of leafy vegetables and blood pressure

**Fig. 2:**
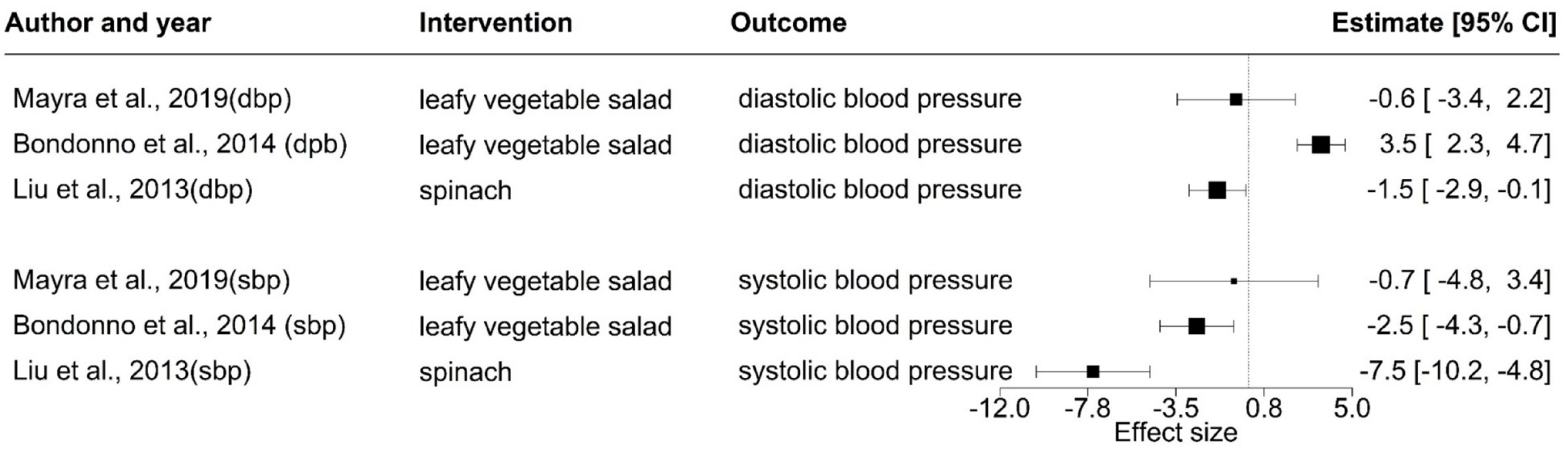
Forest plot indicating the effect of leafy vegetable consumption on systolic and diastolic blood pressure as observed in the RCT’s. dbp, diastolic blood pressure, sbp, systolic blood pressure

Four studies investigated the association between the consumption of leafy vegetables and blood pressure. In one cohort [46] and one RCT [49], reduced risk of hypertension and decreased systolic blood pressure were observed after consuming leafy vegetables. While in two chronic RCTs [51, 57], leafy vegetable consumption had no impact on blood pressure. The effect estimates of the RCTs are shown in Fig. 2.

In the acute crossover RCT by Liu et al. [49], participants consumed 250 g of spinach or a control meal without spinach; blood pressure was recorded pre-meal and every 30 min post-meal for 210 min. A significant (*p* < 0.001) decrease in systolic blood pressure over the 210 min was observed. The highest mean difference of 7.5 mm Hg (Fig. 2) in systolic blood pressure between the control and the intervention group was observed at 120 min post-meal. Although the intervention group had a lower diastolic blood pressure than the control group 120 min post-meal, this was not statistically significant.

No significant difference in diastolic or systolic blood pressure was observed in the crossover RCT by Bondonno et al. [57]. The participants in the intervention group consumed 370 g/day of leafy vegetables, and those in the control group were required to restrict the intake of leafy vegetable salad for seven days; their home blood pressure was monitored each day, ambulatory blood pressure and the office blood pressure was monitored at baseline and after the intervention. The diastolic blood pressure in the intervention group increased by 1.6 mm Hg while that in the control group decreased by 1.9 mm Hg but these differences were not statistically significant. Similarly, in the chronic RCT by Mayra et al. [51], participants (n = 10) consumed 80 g/day of leafy vegetable salad for ten days, and blood pressure was measured on the first and the last day of the intervention. Again, the systolic and diastolic blood pressure change was not statistically significant.

The cohort study included participants (n = 1544) in the third and fourth phases of the “Iranian urban population: Tehran lipid and Glucose Study” [59]. The participants were followed up for three years and were grouped into quartiles based on leafy vegetable intake. The incidence of hypertension in different quartiles was compared. After adjusting for age, weight, smoking, physical activity, and dietary intake of energy, fibre, sodium, potassium, and processed meat, a significant inverse association between the consumption of leafy vegetables and the incidence of hypertension in the odds ratio (OR) was 0.63 with a 95% confidence interval (CI) ranging between 0.41 and 0.98) for participants with the highest intake (a median of 427 g leafy vegetable intake per day) was observed.

### Association between consumption of leafy vegetables and blood glucose

**Fig. 3:**
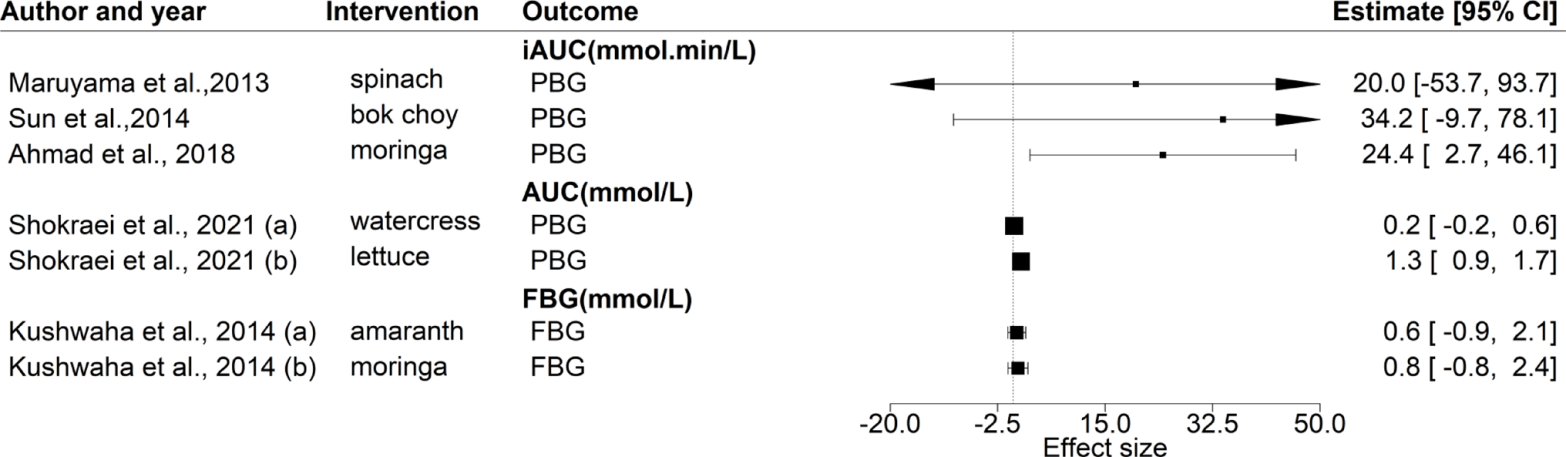
A forest plot of the effect of leafy vegetable consumption on blood glucose as reported in the RCTs. FBG (fasting blood glucose), AUC (area under curve), iAUC (incremental area under curve)

We included six studies investigating the effect of the consumption of leafy vegetables on blood glucose. Consumption of leafy vegetables reduced postprandial and fasting blood glucose in two acute [48] and one chronic RCT [45], respectively. In contrast, two acute RCT [47, 54] found no effect on postprandial blood glucose. A forest plot presenting the effect estimates of the change in blood glucose observed in the RCTs is shown in Fig. 3. No significant association between increased leafy vegetable consumption and the incidence of type 2 diabetes was reported in the cohort study [50].

In the study by Maruyama and colleagues [47], participants consumed 100 g of boiled spinach versus a meal without spinach. Postprandial blood glucose was measured over 240 min, every 30 min for the first hour and every 60 min for the next 3 h. The incremental area under the curve (iAUC) was calculated over 240 min. The peak blood glucose was observed at 30 min, and was higher in the group that consumed spinach than in the control group but was not statistically significant. Additionally, the iAUC of postprandial blood glucose was higher in the spinach group than in the control group but was not statistically significant.

In the acute crossover RCT by Sun et al. [48], a meal with bok choy (*Brassica rapa var. chinensis*) and rice was compared to a meal without bok choy. Blood glucose was measured over 180 min at 15-min intervals for the first hour and 30 min for the following hours. The iAUC was calculated over 120 min. Glucose peaks were observed at 60 and 30 min for the rice only and bok choy-containing meals, respectively. The intervention meals had lower blood glucose peaks than the control. The mean difference between the control and intervention meals was 34.2 mmol. min/L and was significant (*p* < 0.05).

Shokraei and colleagues [58] recruited healthy men in their acute RCT 3-armed crossover study. The participants consumed a meal with either 100 g of watercress, 100 g lettuce, or control without any vegetable but with cellulose. Postprandial blood glucose was monitored every hour for 4 h, and the area under the curve (AUC) for blood glucose concentration was plotted over the four hours. The AUC was 20.1 mmol/L, 21.2 mmol/L, and 21.4 mmol/L for lettuce, watercress, and control, respectively, and were lower when the meal was consumed with lettuce compared to the control (*p* < 0.05) and watercress (*p* < 0.01) meals. The peak blood glucose concentration was observed at 2 h, and at this time, the concentration was lowest in the group that consumed lettuce, followed by the watercress, and highest in the control group. The AUC for blood glucose was significantly higher in the control than in the watercress and lettuce intervention.

The study by Ahmed et al. [54] was an acute crossover RCT in which participants consumed cookies containing 5 g of dried moringa leaves compared to a control group that received plain wheat flour cookies. Postprandial blood glucose was measured every 15 min for 2 h. The peak blood glucose occurred at 30 min and was significantly lower in the intervention group than in the control group. However, there was no significant difference in the iAUC for postprandial blood glucose between the two groups.

In the chronic RCT by Kushwaha et al. [45], participants (n = 90) were supplemented with 9 g of dried amaranth leaves and 9 g of moringa leaves in their daily diet for three months and were compared to a group with no supplementation. The fasting blood glucose was measured before and after the intervention. The fasting blood glucose of the participants in the amaranth intervention was reduced by 0.6 mmol/ L from baseline, and in the moringa intervention, fasting blood glucose was reduced by 0.8 mmol/ L which was statistically significant (*p* < 0.01). In contrast, the control group had no change in fasting blood glucose.

In the cohort study by Kurotani et al., participants (n = 48,437) who were part of the "Japan Public Health Centre Based Prospective" (JPHC) were followed up for five years. A lower risk of type 2 diabetes in participants at the highest quartile of leafy vegetable intake (47.2 g/day) was observed (OR 0.83, 95% CI 0.62–1.12) and (OR 0.81, 95% CI 0.57–16) in men and women respectively, however, this association was not statistically significant. For low intakes of 4–5 g of leafy vegetables, the OR for the incidence of type 2 diabetes was 1.

### Association between consumption of leafy vegetables and blood triglycerides

The acute RCT by Maruyama and colleagues [47] also assessed the impact of consumption of spinach on postprandial triglycerides over 240 min. The mean difference between the control and intervention iAUC of blood triglycerides was 15 mmol.min/L. However, this difference was not statistically significant. Similarly, in the study by Shokraei et al. [58], the consumption of lettuce or watercress did not alter the postprandial blood triglycerides.

### Association between consumption of leafy vegetables and central obesity

None of the studies investigating the relationship between consumption of leafy vegetables and the incidence of central obesity or change in BMI was eligible for inclusion.

### Association between consumption of leafy vegetables and metabolic syndrome

No eligible study investigated the relationship between the consumption of leafy vegetables and the incidence of metabolic syndrome.

## Discussion

Previous systematic reviews have demonstrated beneficial effects of consuming vegetables on the incidence of MetS and its symptoms including a reduced risk of type 2 diabetes and hypertension [60, 61]. However, few studies have conducted subgroup analyses specifically on leafy vegetables, despite their widespread consumption. This is one of the few reviews that investigate the impact of leafy vegetable consumption on the incidence of MetS and its symptoms in cohort and randomised control trials in participants free of any MetS symptoms. Although only ten studies were included, the findings suggest that consuming leafy vegetables may help regulate blood glucose and blood pressure, key symptoms of MetS.

Among the included RCTs, only one reported reduction in blood pressure, whereas the two chronic RCTs found no effect. Previous RCTs also found conflicting results, with some reporting a change in blood pressure [62, 63] while others found no effect [64] after leafy vegetable consumption. Interestingly, when leafy vegetables were consumed longer, as in the cohort study, the onset of hypertension was delayed. Studies have shown that dietary nitrate, present in many leafy vegetables [47], can reduce blood pressure [52, 65–67]. However, a decrease in blood pressure was observed in the acute study with approximately 220 mg of nitrate [68]. In contrast, no significant effect was observed in the two chronic studies with higher amounts, 284 mg/day [48] and 345 mg/day [45] of nitrate. Furthermore, a previous acute study observed a reduction in blood pressure with much lower nitrate levels (143 mg) [69] than in the included studies. Therefore, it could be argued that vegetables may cause several acute drops in blood pressure, as observed in the acute study. The acute effects cumulatively lead to a decline in blood pressure and, consequently, a reduced incidence of hypertension, as observed in the cohort study. However, these cumulative effects might not have been observed in the chronic studies due to the short duration of the studies (7–10 days). Additionally, leafy vegetables contain other bioactive compounds such as flavonoids, organosulfates, magnesium, and potassium, which are associated with controlling blood pressure [29, 70, 71] and were not accounted for in these studies.

Three studies reported a reduction in fasting and postprandial blood glucose, two reported no effect on postprandial blood glucose, and one reported no association between leafy vegetable consumption and incidence of type 2 diabetes. There was a reduction in postprandial blood glucose after consumption of 120 g of cooked bok choy and reduction in fasting blood glucose after consumption of 9 g/day of dry moringa and 9 g/day dry amaranth. In contrast, in the cohort study included in this review, no effect of leafy vegetables on the incidence of type 2 diabetes was observed. Previous studies have found beneficial effects of leafy vegetable consumption in reducing the incidence of type 2 diabetes. For example, a systematic review by Carter et al. reported that consuming 121.9 g per day of leafy vegetables could decrease the incidence of type 2 diabetes by 14% [63]. Additionally, in a previous cohort study by Villegas et al., [72] and a more recent study by Pokharel et al., [73], the consumption of green leafy vegetables was inversely associated with the incidence of type 2 diabetes.

The decrease in blood glucose after leafy vegetable consumption can be attributed to their high fibre content. Fibre causes a reduction in blood glucose by forming gels, which attenuate the increase in postprandial blood glucose and increase insulin sensitivity [32, 74]. Interestingly, the study by Maruyama et al. [47] found no change in postprandial blood glucose, although the intervention had more fibre (6.6 g) than the study by Sun et al. [48], which provided only 1 g of fibre, yet a significant change in postprandial blood glucose was observed. Additionally, in the study by Shokraei et al. [75], the lettuce had an equivalent amount of fibre to the cellulose provided as the control, yet a difference in postprandial response was observed. This suggests that other factors beyond fiber content may play a role in blood glucose regulation. Leafy vegetables also contain bioactive compounds such as flavonoids and phenolic acids that have been found to attenuate postprandial blood glucose response by inhibiting amylolytic enzymes [76], inhibiting glucose transporters [69, 70] and reducing insulin resistance [77]. Therefore, it is possible that these bioactive compounds contributed to the observed differences in blood glucose.

Only two RCTs investigated the impact of leafy vegetable consumption and postprandial blood triglycerides and found no effect. These results agree with a cross-sectional study by Yuan et al. [78], which found no association between vegetable consumption and blood triglycerides. However, other studies have found an inverse association between consuming fruits and vegetables and blood lipids [79, 80].

As reported in two acute RCTs, adding a leafy vegetable did not affect the postprandial HDL-cholesterol [58, 81]. A previous study reported an association between increased consumption of vegetables and increased HDL cholesterol [82]; additionally, diets high in vegetables, such as Mediterranean diets, are associated with increased HDL cholesterol [83]. The mechanism through which these diets impact HDL-cholesterol has not been well understood.

No study on the association between consumption of leafy vegetables and MetS was eligible for inclusion. The cohort study by Hosseinpour et al. almost met our inclusion criteria, but the participants were less than 18 years old. In their cohort study, Hosseinpour and colleagues [84] followed up children and adolescents for three years. They found that consuming at least 27 g of leafy vegetables per week significantly reduced the risk of MetS and its symptoms. This is an achievable amount to consume in a week since it is less than a portion of vegetables. However, the study participants were children, and the amount required to cause such an effect might differ in adults; hence there is a need to investigate this further. Additionally previous research [64] has shown that combining vegetables can produce synergistic, antagonistic, or additive effects, further complicating the interpretation of results. Differences in meal composition across studies could also have influenced the intervention response [85, 86]. Interestingly, in the study by Shokraei et al., while the control meal, the lettuce-containing meal, and the watercress-containing meal had similar compositions aside from the vegetable, each resulted in a different postprandial blood glucose response.

Another important factor is the method of leafy vegetable preparation, which differed across studies. Cooking, for instance, can lead to the loss of essential nutrients such as vitamins [61], as well as phytochemicals like polyphenols [62] and nitrates [63], potentially affecting the outcomes.

The primary aim of this review was to determine whether the consumption of leafy vegetables can reduce the incidence of MetS and to explore their potential role in disease prevention. Previous studies involving participants with MetS symptoms have demonstrated beneficial effects of leafy vegetables in regulating parameters associated with MetS such as blood pressure and blood glucose and their incidence [60, 61]. However, in this review, we excluded studies involving participants with existing MetS symptoms to investigate potential mechanisms without the confounding effects of pre-existing conditions. By focusing on healthy individuals, we aimed to understand how leafy vegetables contribute to maintaining health before the onset of MetS, aligning with dietary recommendations that advocate for their consumption as a preventive measure [87].

## Conclusion

The evidence from this review indicates that leafy vegetables may benefit blood glucose and blood pressure control. However, the evidence is insufficient to recommend leafy vegetables to prevent MetS. Despite the advocacy to increase the consumption of vegetables and fruits to prevent chronic illnesses, there is a lack of well-designed cohort and RCT trials investigating the effect of various types or subgroups of vegetables on the incidence of MetS and its symptoms. This is important as it helps deliver more precise nutrition messages to the public. From this review, we cannot determine whether leafy vegetables are superior to other vegetables in protecting against MetS or any of its symptoms. We suggest that vegetables should be categorised into different subgroups in future studies. This will enable ascertaining which subgroups bring out the most benefits. We also recommend that more RCTs involving healthy humans with different types of vegetables be conducted to ascertain the cause-and-effect relationship with MetS and its symptoms.

## Electronic supplementary material

Below is the link to the electronic supplementary material.

Supplementary Material 1

## Data Availability

This review was registered in the PROSPERO (International prospective register of systematic reviews) database under the number (CRD42020201925).
