# Supplementary material for: Association between leafy vegetable consumption and incidence of metabolic syndrome and its symptoms: a systematic review of prospective cohort and randomised control trials
Source: Eur J Nutr. 2025 Jul 5;64(5):233. doi: 10.1007/s00394-025-03750-6 (PMC12228594; doi:10.1007/s00394-025-03750-6)
Supplement: Supplementary file 1 — Supplementary Material 1 [file 394_2025_3750_MOESM1_ESM.docx]

**Supplementary materials**

**Association between leafy vegetable consumption and incidence of metabolic syndrome and its symptoms: A systematic review of prospective cohort and randomised control trials**

Esther N. Muriuki ^1,2^, Begum Celik ^1^, Gunter G. C. Kuhnle ^1^, Charlotte. E. Mills ^1*^

^1^ Department of Food and Nutritional Sciences, University of Reading, Whiteknights, Harry Nursten Building, Reading RG6 6AP, UK.

^2^ Department of Food Science, Meru University of Science and Technology, Meru-Maua Road, Meru, Kenya.

^*^Email of the corresponding author: *c.e.mills@reading.ac.uk*

Gunter G. C. Kuhnle, Orcid ID: 0000-0002-8081-8931

Charlotte. E. Mills, Orcid ID: 0000-0002-8313-3700

**Table 1: Details of the search strategy**

|  |  | **Search terms** |
| --- | --- | --- |
| **Exposures** | **Exposure 1** | Leaf* OR leaves |
|  | **Exposure 2** | Vegetable* OR plant* OR green* OR amaranth OR brassica* OR cabbage* OR apium OR celery OR “Ceylon spinach” OR spinach OR “spinacia oleracea” OR “pak choi” OR “bok choy” OR cress OR cruciferae OR kale OR lettuce* OR vigna OR blackeyed OR “beta vulgaris” OR chard* OR nasturtium OR watercress OR rocket OR cauliflower OR broccoli OR cowpea* “solanum nigrum” OR “black nightshade” OR “cleome gynandra” OR “Corchorus olitorius” OR pigweed OR “Cucurbita maxima” OR nightshade OR “spider plant” OR “gynandropsis gynandra” OR “jute mallow” OR solanum OR curcubita OR cochorus OR “citrullus lanatus” OR “Talinum triangulare” OR “xanthosoma sagittifolium” OR Abelmoschus OR bidens OR Moringa OR cleome OR mormodica OR “ipomoea batatas” OR “ocimum gratissimum” OR “vernonia amygdalina” |
| **Study outcomes** | **Metabolic syndrome (outcome 1)** | Metabolic syndrome OR “insulin resistance syndrome” OR “syndrome X” OR “plurimetabolic syndrome” OR “Reaven syndrome” OR “dysmetabolic syndrome X” OR “MetS” OR “Metabolic X Syndrome” OR “Dysmetabolic Syndrome” OR “Metabolic Cardiovascular Syndrome” OR “Insulin Resistance Syndrome X” |
|  | **Hypertension (outcome 2)** | hypertens* OR blood pressure |
|  | **Central obesity (outcome 3)** | “body mass index” OR “adiposity” OR “abdominal fat” OR “waist circumference” OR BMI OR “abdominal obesity” OR  "obesity, abdominal" OR "central obesity" OR “truncal obesity” OR “trunk obesity” |
|  | **Hyperglycaemia** **(outcome 4)** | Hyperglycaemia* OR hyperglycemia* OR hyperglycaemia OR hyperglycemia OR “blood glucose” OR “insulin resistance” OR “fasting glucose” OR “Fasting blood glucose” OR “high blood glucose” OR “glucose intolerance” OR “Fasting plasma glucose” |
|  | **Dyslipidemia (raised triglycerides or reduced HDL cholesterol) (outcome 5)** | Triglyceride* OR HDL cholesterol OR low-density lipoprotein cholesterol OR LDL cholesterol OR hyperlipidemia* OR dyslipidemias OR lipoprotein OR LDL OR HDL OR High density lipoprotein OR hypertriglycerid* OR hyperlipidaemia* OR hyperlipidemia* OR dyslipidaemia* OR dyslipidemia* OR hypercholester* OR dyslipidemia* OR dyslipidaemia* |
| **Study designs** | **Terms 1** | Trial* OR study OR studies |
|  | **Terms 2** | Randomi?ed control OR cohort OR clinical OR longitudinal OR prospective* OR random |

The search was performed as follows

#1. Exposure 1 AND exposure 2

#2. Outcome 1 OR outcome 2 OR outcome 3 OR outcome 4 OR outcome 5

#3.Terms 1 AND terms 2

# 1 and # 2 and # 3 (above numbers)

**Table 2: Risk of bias Assessment of the RCT studies**

| **References** | **Randomisation process** | **Bias arising from period and carry-over effects** | **Deviations from the intended intervention** | **Missing outcome data** | **Measurement of the outcome** | **Selection of the reported results** | **Overall quality** |
| --- | --- | --- | --- | --- | --- | --- | --- |
| Liu et al., 2013 [1] | Some concerns | Low risk | Low risk | Low risk | Low risk | Low risk | Medium risk |
| Bondonno et al., 2014 [2] | Some concerns | Low risk | Low risk | Low risk | Low risk | Low risk | Medium risk |
| Mayra et al. 2019 [3] | Some concerns | Low risk | Low risk | Low risk | Low risk | Low risk | Medium risk |
| Sun et al.,2014 [4] | Some concerns | Low risk | Low risk | Low risk | Low risk | Low risk | Medium risk |
| Shokraei et al., 2021 [5] | Some concerns | Low risk | Low risk | Low risk | Low risk | Low risk | Medium risk |
| Ahmad et al., 2018 [6] | High risk | Low risk | Low risk | Low risk | Low risk | Low risk | High risk |
| Maruyama et al. 2013 [6] | High risk | Low risk | Low risk | Low risk | Low risk | Low risk | High risk |
| Kushwaha et al.,2014 [7] | High risk | Low risk | Low risk | Low risk | Low risk | Low risk | High risk |

**Table 3: Risk of bias of cohort studies**

| **Selection** | | | **Comparability** | | | **Outcome** | | | **Score** |
| --- | --- | --- | --- | --- | --- | --- | --- | --- | --- |
| **Reference** | **Representativeness of the exposed cohort** | **Selection of the non-exposed cohort** | **Ascertainment of exposure** | **Demonstration that outcome of interest was not present at the start of the study** | **Comparability of cohorts based on the design or analysis** | **Assessment of outcome** | **Follow up long enough for outcomes to occur** | **Adequacy of follow up** |  |
| Golzarand et al., 2016 [8] | Cluster random sampling was used to select participants from the community ★ | Yes★ | Trained dietitians assessed dietary intake using a validated semi-quantitative food frequency questionnaire (FFQ) ★ | Yes★ | The study controlled for age, sex, weight, smoking, education, and physical activity★ study also controlled for dietary intake of energy, fiber, sodium, potassium and processed meat★ | A trained physician did an independent assessment of blood pressure ★ | Yes ★ (3 years) | Data for 1,544 participants was available at baseline and were followed up for 3 years ★ | 9/9 |
| Kurotani et al.,2013 [9] | Participants were chosen from 11 public health centers ★ | Yes ★ | Participants completed a self-administered FFQ | Yes★ | Multiple logistic regression. The study adjusted for age, BMI, smoking status, alcohol consumption, leisure-time activity, history of hypertension, coffee consumption, family history of diabetes, Mg intake, Ca intake, and energy intake ★★ | Self-reported | Yes★ | Study does not give details of follow up | 6/9 |

A star (★) is equal to one score.

1. Liu, A.H., et al., *Effects of a nitrate-rich meal on arterial stiffness and blood pressure in healthy volunteers.* Nitric Oxide - Biology and Chemistry, 2013. **35**: p. 123-130.

2. Bondonno, C.P., et al., *Short-term effects of a high nitrate diet on nitrate metabolism in healthy individuals.* Nutrients, 2015. **7**(3): p. 1906-1915.

3. Mayra, S.T., C.S. Johnston, and K.L. Sweazea, *High-nitrate salad increased plasma nitrates/nitrites and brachial artery flow-mediated dilation in postmenopausal women: A pilot study.* Nutrition Research, 2019. **65**: p. 99-104.

4. Sun, L., et al., *Effect of chicken, fat and vegetable on glycaemia and insulinaemia to a white rice-based meal in healthy adults.* European Journal of Nutrition, 2014. **53**(8): p. 1719-1726.

5. Shokraei, S., et al., *The acute effect of incorporating lettuce or watercress into a moderately high-fat meal on postprandial lipid, glycemic response, and plasma inflammatory cytokines in healthy young men: a randomized crossover trial.* Lipids Health Dis, 2021. **20**(1): p. 66.

6. Maruyama, C., et al., *Effects of Green-Leafy Vegetable Intake on Postprandial Glycemic and Lipidemic Responses and alpha-Tocopherol Concentration in Normal Weight and Obese Men.* Journal of Nutritional Science and Vitaminology, 2013. **59**(4): p. 264-271.

7. Kushwaha, S., P. Chawla, and A. Kochhar, *Effect of supplementation of drumstick (Moringa oleifera) and amaranth (Amaranthus tricolor) leaves powder on antioxidant profile and oxidative status among postmenopausal women.* J Food Sci Technol, 2014. **51**(11): p. 3464-9.

8. Golzarand, M., et al., *Consumption of nitrate-containing vegetables is inversely associated with hypertension in adults: a prospective investigation from the Tehran Lipid and Glucose Study.* Journal of Nephrology, 2016. **29**(3): p. 377-384.

9. Kurotani, K., et al., *Vegetable and fruit intake and risk of type 2 diabetes: Japan Public Health Center-based Prospective Study.* British Journal of Nutrition, 2013. **109**(4): p. 709-717.
